# An Atypical Presentation of Allergic Bronchopulmonary Aspergillosis

**DOI:** 10.7759/cureus.97627

**Published:** 2025-11-24

**Authors:** Evelien Van Gogh, Abraham Van Poppel

**Affiliations:** 1 Pulmonology, University Hospital of Antwerp, Antwerp, BEL

**Keywords:** abpa, aspergillus species, eosinophilic asthma, pleural effusions, pulmonary aspergillosis

## Abstract

Allergic bronchopulmonary aspergillosis (ABPA) is an immune-mediated hypersensitivity reaction to *Aspergillus fumigatus*, usually occurring in patients with asthma or cystic fibrosis. Presentation with lobar atelectasis or pleural effusion is rare and can mimic malignancy. We report a 57-year-old man without pre-existing lung disease who presented with cough and progressive dyspnea unresponsive to antibiotics. Imaging showed left upper lobe atelectasis with pleural effusion and subcarinal lymphadenopathy. Bronchoscopy revealed complete obstruction of the left upper lobe bronchus by thick mucus plugs; histopathology and culture confirmed *A. fumigatus*. Laboratory testing showed marked eosinophilia, elevated total immunoglobulin-E (IgE), and positive *A. fumigatus* precipitins, findings compatible with the diagnosis of ABPA. Treatment with oral prednisolone and itraconazole led to full clinical and radiological recovery, with sustained remission under inhaled corticosteroid/long-acting β₂-agonist therapy. This case highlights an uncommon presentation of ABPA with pleural effusion and lobar collapse and emphasizes the importance of considering ABPA in the differential diagnosis of unexplained or eosinophilic pleural effusions.

## Introduction

Allergic bronchopulmonary aspergillosis (ABPA) is a chronic airway disorder caused by an immune-mediated hypersensitivity reaction to *Aspergillus *spores, most commonly *A. fumigatus*. The disease results from colonization of the airways by the fungus, leading to allergic inflammation, mucus impaction, and bronchial obstruction. It most frequently occurs in patients with asthma or cystic fibrosis [1,2].

Its clinical and radiological presentation is highly variable, ranging from asymptomatic cases to severe, uncontrolled asthma with extensive radiographic abnormalities. Cough and dyspnea are the most frequently reported symptoms (99%), while hemoptysis is also common, occurring in approximately 41% of cases [1]. A characteristic feature of ABPA is the formation of mucus plugs rich in eosinophils due to colonization of the airways by the fungus, which can cause fixed airflow obstruction, with lung collapse and bronchiectasis. Radiographically, consolidations are the most frequently observed finding, seen in up to 91% of patients with ABPA. Bronchiectasis is identified on imaging in approximately 60% of cases. Pleural effusion, however, is a less frequently reported presentation of ABPA [1-3]. We present a case of pleural effusion and partial lung collapse as the initial presentation of ABPA.

## Case presentation

A 57-year-old male, non-smoker, with a history of transurethral resection of the prostate (TURP) and chronic sinusitis, and with no prior pulmonary history, nor any known tuberculosis or other previous infections, suffered from a persistent cough and sore throat for a few weeks. He was referred to the pulmonary department by his general practitioner due to a lack of improvement of symptoms under amoxicillin-clavulanate therapy and the development of progressive dyspnea. He had no hemoptysis, no fever, weight loss, or night sweats. On examination, he had dullness to percussion over the left side of the chest, with normal vital signs. No clinical abnormalities of the chest wall were observed. Pulmonary function testing showed a severely restrictive pattern, with both forced vital capacity (FVC) and total lung capacity (TLC) reduced to about 40% of the predicted values. There were no signs of airflow obstruction.

**Figure 1:**
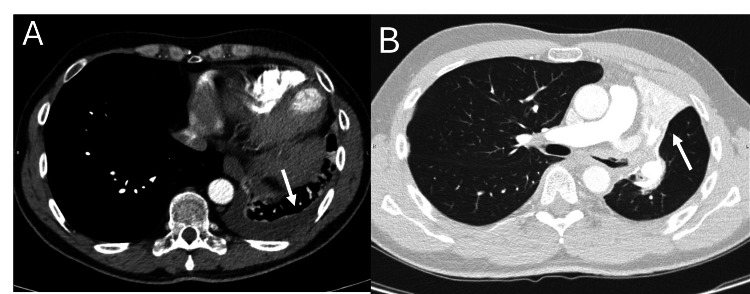
Computed tomography of the chest at first presentation. (A) Computed tomography of the chest shows left-sided pleural effusion (arrow). (B) The lung window shows complete atelectasis of the left upper lobe and lingula (arrow) due to obstruction of the left upper lobe bronchus.

A computed tomography of the chest revealed extensive atelectasis of the left upper lobe and lingula caused by obstruction of the left upper lobe bronchus (Figure 1B). There were also subcarinal lymph node enlargement and a left-sided pleural effusion in the left lower lobe (Figure 1A). There was a suspicion of malignancy. 

An urgent bronchoscopy showed complete blockage of the left upper lobe bronchus by thick, purulent mucus plugs. A lengthy procedure was required to remove as much of the mucus as possible. However, the upper lobe bronchus remained inaccessible, and biopsies could not yet be obtained. Laboratory findings showed numerous fungal hyphae with acute inflammation, but no signs of malignancy.

The patient was, therefore, referred to a tertiary care center for further evaluation. A repeat bronchoscopy revealed persistent thick mucus plugs, which were successfully removed, permitting visualization of the mucosa of the upper lobe and lingula, which appeared very fragile and bled easily. Bronchial biopsies again showed no evidence of malignancy, though abundant fungal hyphae were identified and culture showed *Aspergillus fumigatus*.

Blood tests revealed marked eosinophilia with a total eosinophil count of 1500 cells/µL, a high serum immunoglobulin-E level (IgE) (1432 IU/mL, normal <114), and an elevated *A. fumigatus* specific IgE level (6.46 kilo-units of antibody per liter, normal <0.35). Serum precipitins to *A. fumigatus* were positive. Subsequent pulmonary function testing showed resolution of the restrictive pattern with the appearance of an obstructive pattern, compatible with underlying asthma. Consequently, inhaled corticosteroid and long-acting β₂-agonist (ICS/LABA) therapy was started.

Finally, the patient was diagnosed with allergic bronchopulmonary aspergillosis (ABPA) with underlying eosinophilic asthma. Treatment for ABPA was started with oral prednisolone and itraconazole, the latter added because of the extent of pulmonary involvement.

**Figure 2:**
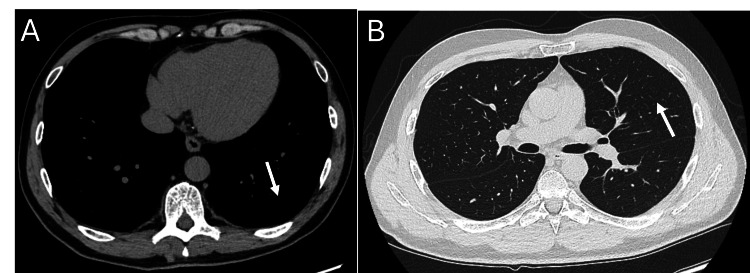
Computed tomography of the chest after three-month treatment. (A) Computed tomography of the chest shows complete resolution of the left-sided pleural effusion (arrow). (B) The lung window shows complete re-expansion of the retro-obstructive atelectasis in the lingula (arrow) and clearance of endobronchial mucus impaction within the lingular bronchi.

At the three-month follow-up, the patient's pulmonary symptoms resolved, accompanied by progressive recovery on radiological and pulmonary function testing. Imaging confirmed gradual resolution of mucus impaction and re-expansion of the previously collapsed lung segments and resolution of the pleural effusion (Figures 2A, 2B). 

Treatment with itraconazole and prednisolone was successfully discontinued after three months. Maintenance therapy with ICS/LABA was continued. At the six-month, 12-month, and two-year follow-up, the patient remained clinically stable with no signs of recurrence.

## Discussion

Aspergillus is an opportunistic pathogen that can trigger hypersensitivity reactions in immunocompetent individuals. The underlying mechanism is not fully understood, but it is thought that inhaled Aspergillus spores bind to pulmonary surfactant, which then forms hyphae. This process leads to the release of various interleukins and an increase in IgE, IgG, and IgA antibodies, resulting in impaired phagocytosis and reduced mucociliary clearance [1,2]. Mucus impaction may lead to complete bronchial obstruction and lobar collapse, as observed in this case.

Pleural effusion in ABPA is believed to occur through two main mechanisms. First, a mechanical effect of atelectasis may cause fluid accumulation within the pleural space [1,3]. Second, the type 2 helper T-cell-mediated inflammatory response (characterized by cytokine release and possible fungal translocation into the pleural cavity) may trigger a local pleural inflammatory reaction, resulting in exudative effusion [3].

In this case, the patient developed progressive dyspnea due to atelectasis from mucus impaction and obstruction of the left upper lobe bronchus, combined with pleural effusion. These radiological abnormalities and subcarinal lymphadenopathy initially raised suspicion of malignancy. Histopathological examination, however, demonstrated numerous fungal hyphae without malignant cells, leading to the diagnosis of ABPA.

The patient met the revised International Society for Human and Animal Mycology (ISHAM) diagnostic criteria for ABPA [4], including elevated total IgE, positive *Aspergillus fumigatus* IgE, eosinophilia >500, high Aspergillus IgG, and radiological evidence. Although he had no prior history of asthma, underlying eosinophilic asthma was later identified as the likely predisposing factor.

Diagnosing ABPA in the context of lung collapse and pleural effusion is particularly challenging, especially in patients without prior respiratory disease or typical imaging findings.

Pleural puncture can be helpful for diagnostic clarification. Several case reports describe the pleural fluid as exudative, most commonly eosinophilic, although neutrophilic and lymphocytic profiles have also been reported. An acidic pH has likewise been described in some cases [3]. In our patient, pleural puncture was not performed, as the diagnosis was established early based on bronchoscopic findings.

Bronchoscopy is crucial for both diagnosis and therapy, allowing visualization and removal of mucus plugs and collection of diagnostic material. In this case, repeated bronchoscopic procedures were required to remove dense sputum plugs and confirm the presence of fungal hyphae.

Systemic corticosteroids remain the cornerstone of ABPA treatment, suppressing inflammation and hypersensitivity. Itraconazole was added to reduce fungal burden and achieve a steroid-sparing effect. This combined regimen led to marked clinical, radiological, and functional improvement, with complete re-expansion of the affected lung [1].

Pleural effusion in ABPA has been reported only rarely. A PubMed search revealed a limited number of cases, the most recent published in 2020, with others dating back to 1981, the early 2000s, and 2011. In all reports, ABPA was not initially suspected due to its atypical and uncommon presentation. The diagnosis was most often established based on elevated serum IgE levels, positive precipitating serum antibodies to *A. fumigatus*, and/or a positive immediate skin test to *A. fumigatus* [3,5-7]. In one case, the diagnosis was made following bronchoscopy with culture confirming *A. fumigatus* [8].

## Conclusions

In conclusion, this case demonstrates that ABPA can rarely present with lobar atelectasis and pleural effusion, even in patients without known chronic lung disease. Early bronchoscopy is essential for diagnosis, airway clearance, and exclusion of malignancy, while prompt corticosteroid and antifungal therapy helps prevent long-term complications. Although pleural effusion is an uncommon manifestation, ABPA should be considered in the differential diagnosis of unexplained or eosinophilic pleural effusions, particularly in patients with asthma or eosinophilic airway disease.
